# AgBiS_2_ as a low-cost and eco-friendly all-inorganic photovoltaic material: nanoscale morphology–property relationship†

**DOI:** 10.1039/c9na00505f

**Published:** 2019-12-12

**Authors:** Ming-Gang Ju, Jun Dai, Liang Ma, Yuanyuan Zhou, Xiao Cheng Zeng

**Affiliations:** Department of Chemistry, University of Nebraska-Lincoln Lincoln Nebraska 68588 USA xzeng1@unl.edu; Southeast University Nanjing 211189 China; School of Engineering, Brown University Providence Rhode Island 02912 USA; Department of Chemical & Biomolecular Engineering, Department of Mechanical & Materials Engineering, University of Nebraska-Lincoln Lincoln Nebraska 68588 USA

## Abstract

Solar cells made of low-cost solution-processed all-inorganic materials are a promising alternative to conventional solar cells made of high-temperature processed inorganic materials, especially because many high-temperature processed inorganic materials contain toxic element(s) such as lead or cadmium (*e.g.*, CsPbI_3_ perovskite, PbS, CdTe and CdS(Se)). AgBiS_2_ nanocrystals, consisting of earth-abundant elements but without lead and cadmium, have already emerged as a promising candidate in high-performance solar cells. However, the nanoscale morphology–optoelectronic property relationship for AgBiS_2_ nanocrystals is still largely unknown. Herein, we investigate the electronic properties of various AgBiS_2_ nanocrystals by using first-principles computation. We show that the optoelectronic properties of bulk AgBiS_2_ are highly dependent on the M–S–M–S– (M: Ag or Bi) orderings. Moreover, because Ag–S–Ag–S– and Bi–S–Bi–S– in AgBiS_2_ bulk crystals contribute respectively to the valence band maximum and conduction band minimum, these unique chemical orderings actually benefit easy separation of mobile electrons and holes for photovoltaic application. More importantly, we find that AgBiS_2_ nanocrystals (NCs) can exhibit markedly different optoelectronic properties, depending on their stoichiometry. NCs with minor off-stoichiometry give rise to mid-gap states, whereas NCs with substantial off-stoichiometry give rise to many deep defect states in the band gap, and some NCs even show metallic-like electronic behavior. We also find that the deep-defect states can be removed through ligand passivation with optimal coverage. The new insights into the nanoscale morphology–optoelectronic property relationship offer a rational design strategy to engineer the band alignment of AgBiS_2_ NC layers while addressing some known challenging issues inherent in all-inorganic photovoltaic materials.

## Abstract 2

AgBiS_2_ nanocrystals can exhibit markedly different optoelectronic properties depending on their stoichiometry, and the optoelectronic properties can be tuned through ligand passivation.

## Graphical abstract

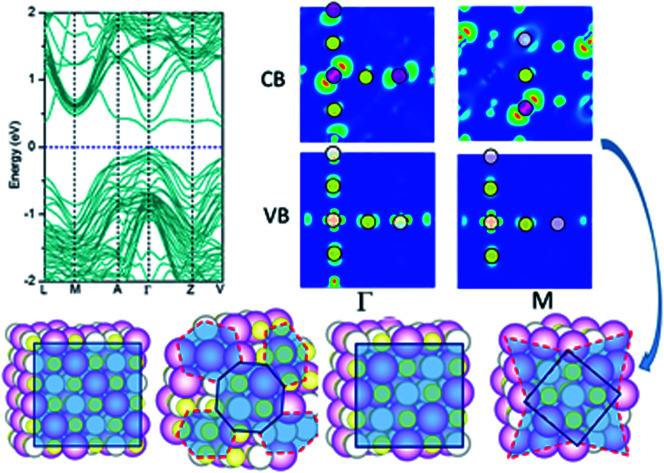

## Introduction

Recently, solution-processed inorganic photovoltaic (PV) materials have attracted considerable attention primarily due to their significantly reduced manufacturing cost compared to that for producing conventional inorganic PV materials such as Si and GaAs. The most studied solution-processed inorganic PV materials with respectable power conversion efficiency (PCE) include lead sulfide (PbS),^1,2^ cadmium telluride (CdTe),^3^ antimony sulfide-selenide (Sb_2_(S,Se)_3_),^4^ and copper zinc tin sulfide–selenide (CZT(S,Se)).^5^ However, PbS and CdTe contain a large portion of toxic elements (Pb or Cd), and the processing of Sb_2_(S,Se)_3_ and CZT(S,Se) generally involves an expensive, complex, and high-temperature selenization/sintering step.^4–6^ These restrictions have held back large-scale commercialization of these materials. Over the past decade, a rich family of materials called lead-halide perovskites, *e.g.* CH_3_NH_3_PbI_3_ and CsPbI_3_,^7,8^ have emerged and achieved high PV performance.^9–13^ Still, the inclusion of the element Pb in most of these materials is of concern for large-scale PV application.^14–17^ Currently, the development of lead-free perovskites remains a challenging task, as solar cells based on lead-free hybrid organic–inorganic perovskites typically give lower PCE than lead-containing perovskite solar cells and yet still have low stability. Hence, low-cost inorganic PV materials with high stability and environmental friendliness are highly sought.

Nanocrystal PVs have shown major progress due to significant improvement in surface passivation strategies and device structure evolution. Among nanocrystal PVs, lead chalcogenide PbS and PbSe quantum dot solar cells stand out and have respectively achieved a PCE of 11.23% (ref. 2) and 8.2% (ref. 18) due to their favorable bandgaps for light harvesting and favorable charge transport resulting from large exciton Bohr radii. On the other hand, AgBiS_2_ nanocrystals (NCs) have emerged as a candidate lead-free inorganic PV material, because the nanocrystals are composed of non-toxic and earth-abundant elements.^19–21^ Applications of AgBiS_2_ NCs as a solar-cell material were first reported by Bernechea *et al.* in 2016, although the synthesis of AgBiS_2_ bulk and nanocrystal materials has been reported much earlier.^19^ Bernechea *et al.* demonstrated a certified PCE of 6.3% for AgBiS_2_ NCs based solar cells fabricated at the ambient pressure and at temperatures below 100 °C. Note that all elements (Ag, Bi, and S) in AgBiS_2_ NCs are earth-abundant. Although this experiment has suggested AgBiS_2_ NCs as a promising PV-material, the structure–electronic property relationship and structural properties of the nanocrystal are still lacking in the literature. In this study, we performed density functional theory (DFT) calculations on AgBiS_2_ bulk and nanocrystals, and offered explanations for the desirable electronic properties of both bulk and nanocrystals. Knowledge obtained from the computational studies can guide modification of the structural and electronic properties of AgBiS_2_ NCs for achieving better PV functionality.

## Results and discussion

**Fig. 1:**
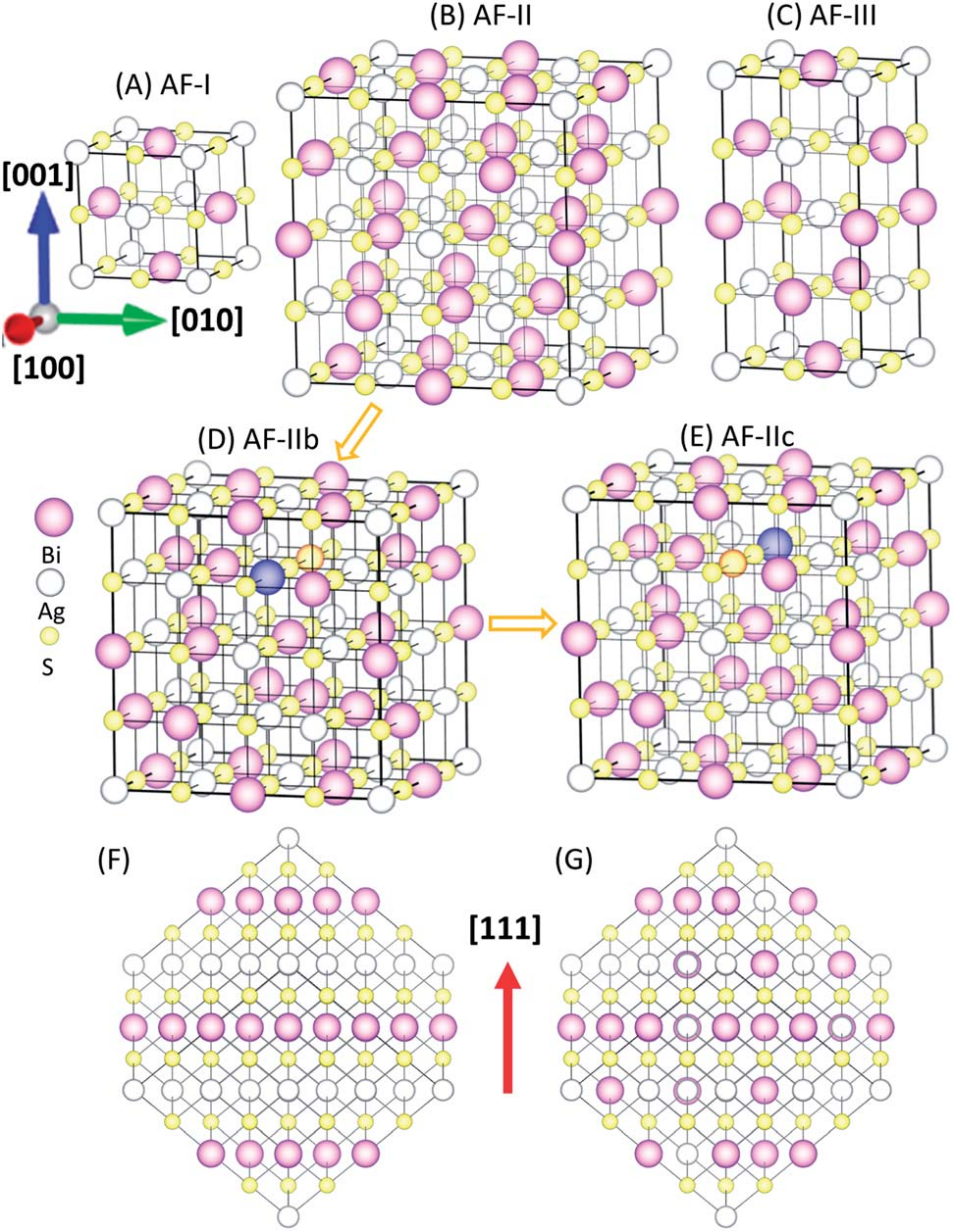
Possible ordered structures of AgBiS_2_: (A) AF-I (space groups: *P*4/*mmm*), (B) AF-II (*R*3̄*m*), (C) AF-III (*I*4_1_/*amd*), (D) AF-IIb (*F*3̄*dm*), and (E) AF-IIc (*C*2/*m*). (F) AF-II viewed in the direction normal to [111]. (G) AF-IIb viewed in the direction normal to [111]. Indigo and orange balls denote the swapped Bi and Ag sites from AF-IIb to AF-IIc.

It is known that connectivity of the Ag–Bi–S network plays an important role in the electronic structures. Hence, the ordering of Ag and Bi atoms needs to be identified. Here, we describe different types of orderings of Ag and Bi ions by using the standard nomenclature of antiferromagnetic (AF) orderings.^22^ The simplest structure is AF-I (see Fig. 1A) with alternating Ag–S and Bi–S planes normal to the [100] direction. AF-III (Fig. 1C) exhibits mixed Ag and Bi atoms in the Ag–S and Bi–S planes of AF-I, and Ag–S–Ag–S– and Bi–S–Bi–S– chains along the *a* and *b* directions. To disrupt these chains, we constructed an interrupted chain model (AF-II) that exhibits the sequence of Bi–S–Ag–S– chains in all three directions (Fig. 1B). AF-IIb also exhibits the sequence of Bi–S–Ag–S– chains in all three directions. AF-II can be viewed as alternating Ag, S and Bi planes normal to the [111] direction (see Fig. 1F), while AF-IIb can be viewed as alternating Ag, Bi and S planes normal to the [111] direction (see Fig. 1G). To swap the Bi and Ag in the second layer of AF-IIb, AF-IIc exhibits two Ag–S–Ag–S– and Bi–S–Bi–S– chains in the crystal, mixed with the interrupted chain. The primitive cells of AF-I, AF-II, AF-IIb, AF-IIc and AF-III are displayed in Fig. S1,† which are, respectively, simple tetragonal (*P*4/*mmm*), hexagonal representation (*R*3̄*m*), fcc (*F*3̄*dm*), monoclinic (*C*2/*m*) and bct (*I*4_1_/*amd*). Energetically, AF-I and AF-III possess higher energy among polymorphs of AgBiS_2_ (Table 1). In particular, AF-II, AF-IIb and AF-IIc possess similar stabilities, while AF-IIb has the lowest energy. The Ag–S–Bi–S– chains in AF-II and AF-IIb are unique, resulting in small energy difference between AF-II and AF-IIb, *i.e.*, Δ*E* ∼ 7 meV per formula unit (f.u.). With the increase of the Ag–S–Ag–S– and Bi–S–Bi–S– chains, the total energy of crystal structures becomes less favorable, suggesting that Ag in the chains strongly perturbs hybridized Bi and S p bands, resulting in shifting states near the Fermi level to lower energy. Fig. S2† displays simulated X-ray diffraction (XRD) patterns of AF-I, AF-II, AF-IIb, AF-IIc and AF-III, as well as experimental XRD patterns. Notably, the simulated XRD patterns of AF-II, AF-IIb and AF-IIc are much closer to the AgBiS_2_ XRD patterns reported in the literature,^19^ suggesting that the crystal structures of AF-II, AF-IIb and AF-IIc are closer to the realistic crystal structure of AgBiS_2_, consistent with the obtained relative stability among the aforementioned five structures.

**Table 1:** Computed relative energies and bandgaps of AgBiS_2_ bulk crystals with different ordering structures. The energy of AF-I is defined as a reference

Sample	AF-I	AF-II	AF-IIb	AF-IIc	AF-III
Δ*E* (eV)	0	−0.377	−0.384	−0.353	−0.242
Bandgap (eV)	0	0.85	1.30	0.80	0.65

Next, we discuss the electronic properties of these compounds and relationship with the orderings of Ag and Bi. Fig. S3–S5† show the projected DOS (PDOS) and band structures of AF-I, AF-II and AF-III. Clearly, AF-I possesses a metallic feature, while AF-II and AF-III exhibit bandgaps of 0.85 and 0.65 eV (the computed bandgaps of the five structures are summarized in Table 1), respectively. For these three inorganic compounds, the proportion of Bi–S–Ag–S– chains increases in the order of AF-I < AF-III < AF-II, and the corresponding bandgaps also increase in the order of AF-I < AF-III < AF-II. The Fermi level of AF-I lies near the rapidly decreasing part of the DOS, mostly contributed by the p orbital of Ag and S in the Ag–S planes. Strong hybridization among p orbitals of Bi and S contributes to the region near the Fermi level. From AF-I to AF-III, the electronic behaviors range from metallic to semiconducting. The DOS around the bandgap edge stems from hybridized states between the Bi 6p orbital and S 3p orbital that are significantly suppressed. The VBM is mostly contributed by Ag 4d, Bi 6s and S 3p orbitals and the CBM is mostly contributed by the Bi 6p orbital and S 3s orbital (see Fig. S5†). For AF-II, there is a wider bandgap of 0.85 eV. The DOS around the VBM and CBM reflects similar contribution with respect to AF-III, besides that the Bi 6s and S 3s orbitals contribute more with respect to other orbitals. The above analysis shows that, with the increase of the interrupted chain Bi–S–Ag–S–, the hybridized states, stemming from the Bi 6p and S 3p orbitals, are suppressed, while the Bi 6s and S 3s orbitals are respectively incorporated in the VBM and CBM, resulting in a relatively wide bandgap for AgBiS_2_.

Both AF-IIb and AF-IIc are semiconductors with bandgaps of 1.3 and 0.8 eV, respectively, although AF-II, AF-IIb and AF-III possess indirect bandgaps. A direct allowed transition is important for the strong absorbance of good PV materials. Interestingly, through swapping Ag and Bi in AF-IIb, AF-IIc possesses a direct bandgap. To illustrate this fundamental structural difference, supercells of AF-IIb and PbS are constructed based on the unit cell of AF-IIc. Note that the structural relationship between PbS and AgBiS_2_ can be viewed as replacement of lead by much less toxic bismuth along with 1^+^ cations such as Ag^+^ through cation mutation, akin to that between A^I^M^II^X_3_ perovskite and A^I^_2_M^I^M^III^X_6_ double perovskite.^23^

**Fig. 2:**
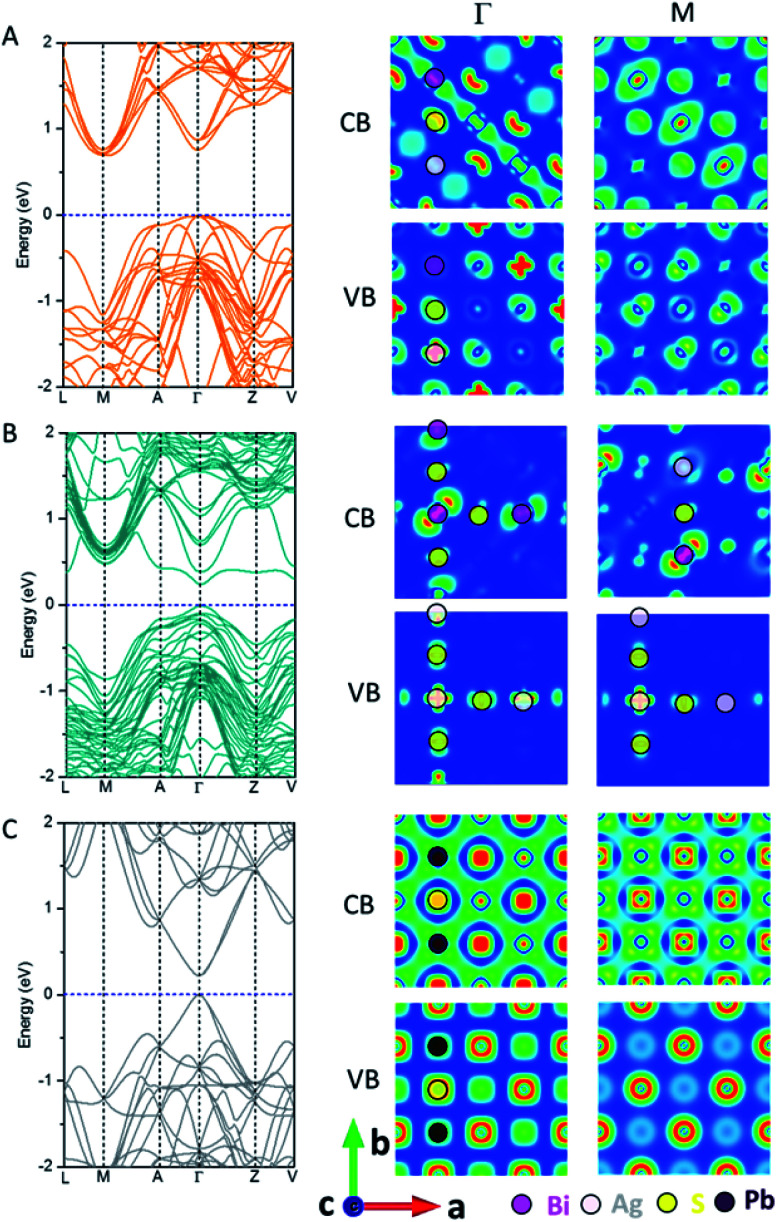
Computed band structure and charge density of the CBM and VBM at *M* and *Γ* points of AF-IIb (A), AF-IIc (B) and PbS (C).

Fig. 2 shows the electronic band structure and charge density distributions of the CBM and VBM at *M* and *Γ* points. It can be seen that PbS and AF-IIc possess a smaller bandgap than AF-IIb. PbS possesses a much wider conduction band and the bandwidth is close to 2.4 eV, arising from the interaction of Pb 6p orbitals and S 3s and 3p orbitals at the *Γ* point, compared to the highly antibonding configuration at the *M* point. In the valence band of PbS, at *M*, the band is mostly localized on S anions, whereas at *Γ*, the strongly directional interaction between S 3p orbitals and the Pb 6s orbitals leads to a much higher valence band, compared to that at *M*. For AF-IIb, the frontier orbitals are attributed to either Ag or Bi, resulting in weak interaction with the nearest-neighbor cation, thereby leading to a more localized and narrow conduction band and a large bandgap. In particular, at *Γ*, the highest valence band is localized only on the Ag cation and S anion, while the lowest conduction band is localized on Bi cations and S anions. At *M*, the highest valence band is mostly localized on S anions while the lowest conduction band is localized on the Bi and S. For the valence band, a stronger directional interaction between S 3p and Ag 4d orbitals at *Γ* leads to a much higher valence band, compared to the weak interaction between Bi 6s and S 3p orbitals at *M*. For the conduction band, at *M*, a stronger interaction between Bi 6p orbitals and S 3s orbitals (compared to moderate interaction between neighbouring Bi 6p orbitals) together with some S 3s orbitals at *Γ* leads to a lower conduction band at *M*. As a result, AF-IIb possesses an indirect bandgap, arising from electronic mismatch between Ag and Bi, whereas AF-IIc possesses a direct bandgap.

On the bulk structure, the difference between AF-IIb and AF-IIc is that only Bi–S–Ag–S– chains exist in AF-IIb whereas both Ag–S–Ag–S– and Bi–S–Bi– chains exist in AF-IIc besides Bi–S–Ag–S– chains. Importantly, the highest valence bands contributed by Ag 4d and S 3p orbitals at *Γ* and *M* points are localized in the Ag–S–Ag–S– chain. However, the valence band is delocalized at *Γ* in all chains, whereas the valence band at *M* is localized on Ag and nearest-neighbour S in the Ag–S–Ag–S– chain, leading to a higher valence band at *Γ*. Analogous to the valence band, the conduction band contributed by Bi 6p and S 3p orbitals is delocalized on the Bi–S–Bi–S– chain at *Γ*, compared to the conduction band at *M*, which is localized on the Bi and nearest-neighbor S, resulting in the lowest conduction band at *Γ*. In sum, the chains Ag–S–Ag–S– and Bi–S–Bi–S– contribute to the VBM and CBM, respectively, due to stronger interaction between Ag 4d and S 3p orbitals and between Bi 6p and S 3p orbitals, with respect to the mixed chain Bi–S–Ag–S–. Fig. S6 and Table S1† show the computed charge distribution and band edge compositions of the VB and CB at *M* and *Γ* points, while Fig. S7† shows the computed DOSs of three compounds, confirming the above analysis. Considering the heavy atom Bi in AgBiS_2_, spin–orbit coupling (SOC) is important. Fig. S8† shows the computed DOS of AF-IIb with consideration of SOC. Clearly, the computed bandgap is narrowed by about 0.3 eV due to the CBM being contributed by Bi 6p orbitals.

**Fig. 3:**
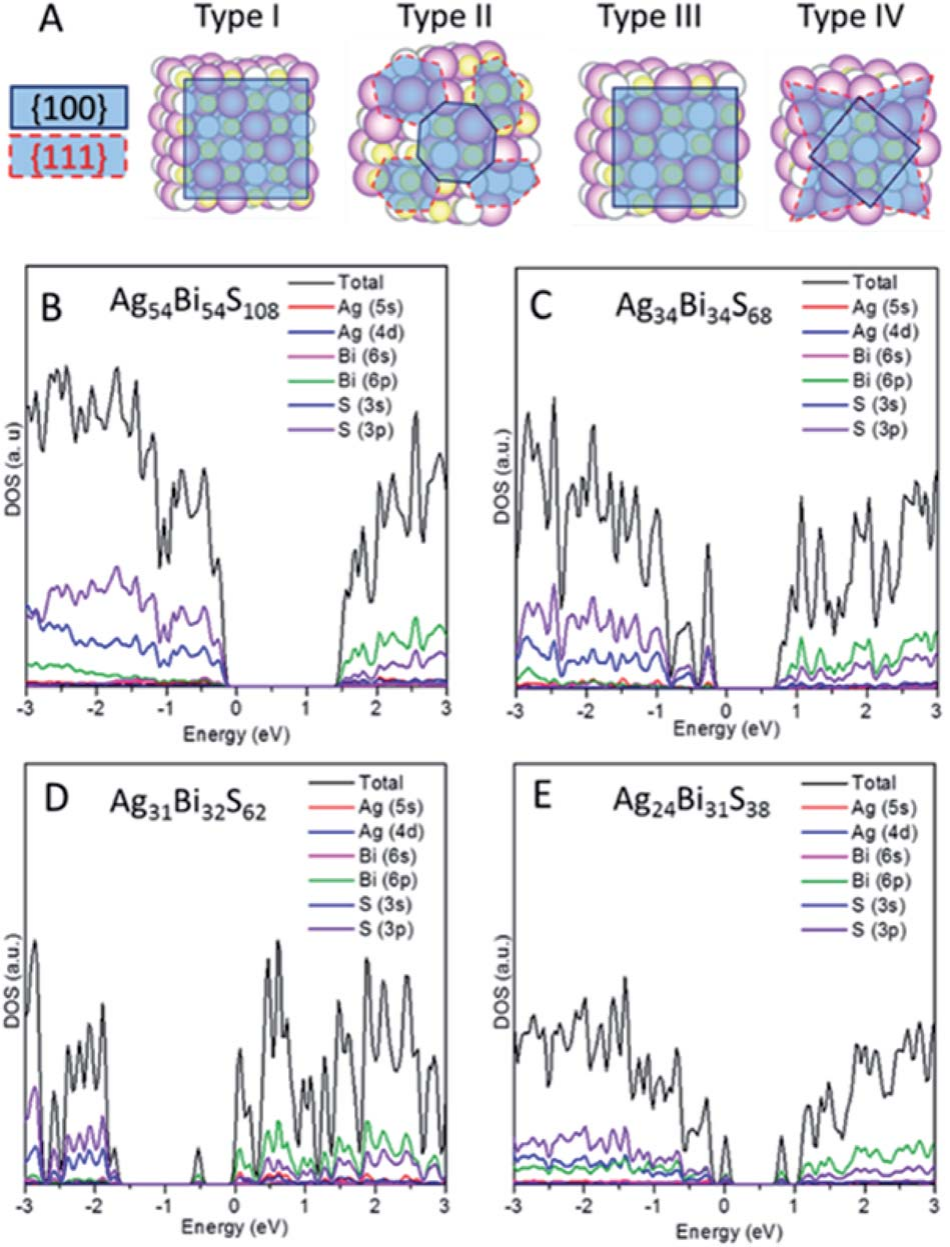
(A) Classification of AgBiS_2_ NCs into four different types (I, II, III, and IV). PDOS of each type of NC: type I Ag_54_Bi_54_S_108_ (B), type II Ag_34_Bi_34_S_68_ (C), type III Ag_31_Bi_32_S_62_ (D) and type IV Ag_24_Bi_31_S_38_ (E).

Note that the (100) and (111) crystalline planes are the dominant facets of PbS NCs.^24,25^ Here, we consider NC shapes of a perfect cube and truncated octahedron with 8-(111) facets. As shown in Fig. 3A, four different types of NCs carved out from the AF-IIb crystal are classified as types I–IV, based on NC shapes and stoichiometry. Types I and III are cube-shaped NCs with the (001) surface planes exposed, whereas types II and IV exhibit faceted cube shapes with both (100) and (111) surface planes exposed. The stoichiometry of types I and II NCs is Ag_*n*_Bi_*n*_S_2*n*_, that of type III NCs is either Ag_*n*_Bi_*m*_S_*n*+*m*+1_ or Ag_*n*_Bi_*m*_S_*n*+*m*−1_ (|*n* − *m*| ≤ 1), and that of type IV NCs is Ag_*m*_Bi_*n*_S_*x*_ (|*m* + *n* − *x*| ≥ 7) due to the richness of either metal or sulphur atoms on the faceted (111) surfaces. The type I and II NCs are stoichiometric NCs while types III and IV are off-stoichiometric NCs.

We first compute the electronic properties of purely stoichiometric NCs. Here, Bi and Ag donate three and one electrons, respectively, while S accepts two electrons in the compounds. We define *R* as (Ag + 3Bi)/2S, an important factor that can be used to tune the properties of AgBiS_2_ NCs. For type I and type II NCs, *R* is fixed at unity due to stoichiometry. The electronic structure shows that type I and type II are semiconductors without having deep defect states (see Fig. 3B, C and S9†). Considering the trend of bandgaps with NC size and shape, type II has narrower bandgaps than type I for a given size, suggesting that charges in the truncated octahedron NCs are less quantum confined than those in the cube NCs (see Fig. S10B†). Moreover, the energy difference between the vacuum level and the Fermi level (*E*_vac_ − *E*_F_) decreases with increasing the size, indicating higher ionization potential for larger NCs (see Fig. S10A†). Fig. 3B and C show the computed DOSs of type I and type II. Type I NCs possess an ideal bandgap, while there are shallow states around the VBM for type II NCs. The VBM of both NCs are mostly contributed by Ag 4d and S 3p orbitals, while CBM of both NCs are mostly contributed by Bi 6p and S 3p orbitals. Analogous to bulk AF-IIc, we consider swapping a Bi and Ag site in NCs. For example, we exchange a Bi and Ag site in type I NC Ag_54_Bi_54_S_108_, resulting in two different NCs (named type 1b and type 1c) due to the Ag and Bi site with respect to the surface. The bandgaps of the two different NCs (type 1b and type 1c) are narrower than those of original (see Fig. S11†). Similar to AF-IIc, the CBM is localized on the Bi–S–Bi–S– chains in NCs, resulting in a narrower bandgap, which would benefit the separation of excitons due to the desired separation of the CBM and VBM.

Next, we focus on off-stoichiometric NCs, types III and IV. Fig. 3D shows the projected DOS of type III NCs (Ag_31_Bi_32_S_62_, *R* = 1.024). Here, a mid-gap state emerges, which has been observed in experiments,^26^ and plays a crucial role in the charge transport of NC films. For type III NCs with different compositions of Ag and Bi, only some NCs possess mid-gap states (see Fig. S12†). In the above example, the mid-gap state of the Ag_31_Bi_32_S_62_ NC is delocalized on the central Ag and nearest-neighbour S and Bi atoms (see Fig. S13A†). Note that all type III NCs with equal composition of Bi and Ag possess mid-gap states, delocalized on the corner Bi and nearest-neighbor atoms for metal-rich type III NCs or delocalized on the corner S and nearest-neighbour atoms for S-rich type III NCs (see Fig. S13B and C†). Interestingly, the delocalized distributions of mid-gap states for NCs with equal and different compositions of Ag and Bi are very different. For the small-sized type III NCs, the DOSs around bandgaps are separated in energy level, akin to the electronic structure of molecules. With relatively large sized type III NCs, *e.g.*, Ag_85_Bi_86_S_172_, the DOSs around gaps are continuous, like a bulk semiconductor.

When the (111) facets are introduced at the corners of type III NCs, off-stoichiometric type IV NCs are formed. Fig. 3E shows an example of type IV NC, Ag_24_Bi_31_S_38_. Unlike the other three types of NCs, type IV NCs show many deep defect states in the bandgap, which can become a recombination centers and are unfavorable to the performance of PV. In particular, for *R* > 1, several discontinuous defect states exist in the bandgaps (see Fig. S14†). For *R* < 1, type IV NCs show metallic-like electronic behaviour, with a dense number of energy states near the Fermi level (see Fig. S15†).

**Fig. 4:**
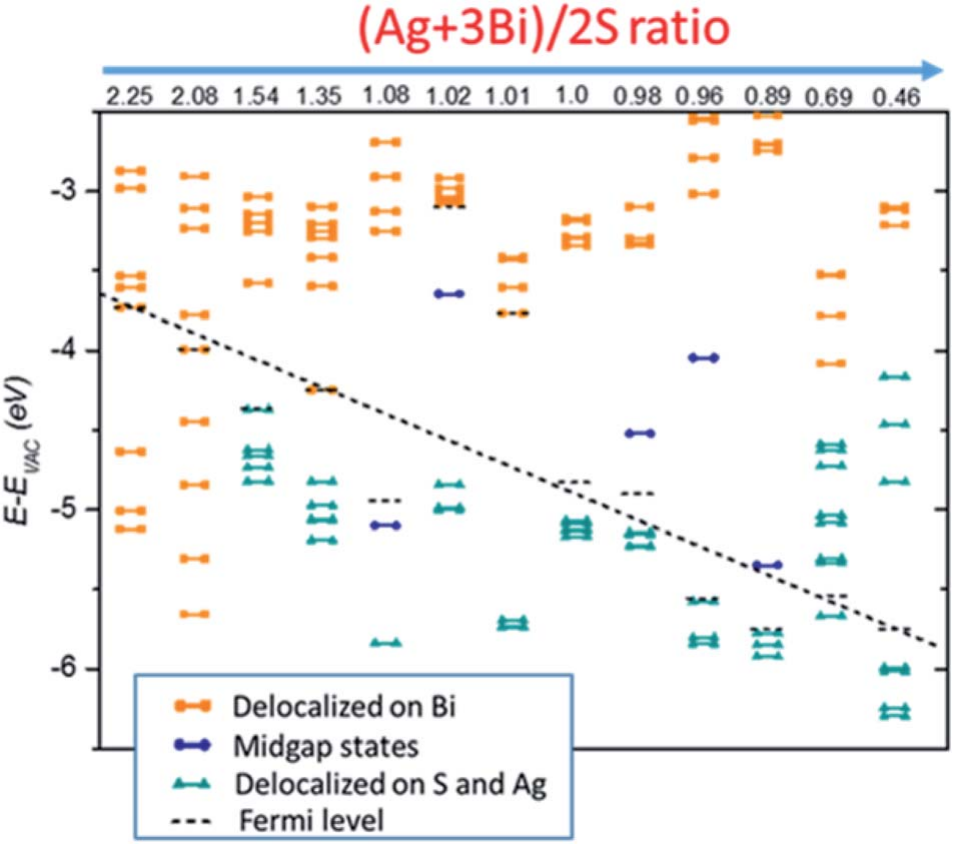
Kohn–Sham energy levels of NCs, shifted with respect to the vacuum level, for NCs with various stoichiometries. Electronic states in orange are mostly delocalized over Bi atoms whereas those in cyan are mostly delocalized over S and Ag atoms, and the states in blue refer to localized mid-gap states. States below the black dashed line are occupied while others are unoccupied in the ground state.

The four case studies shown above indicate that electronic structures are very sensitive to the NC stoichiometry. More comprehensive stoichiometry analyses with *R* values varying from 2.25 to 0.46 are performed. As shown in Fig. 4, type I and II stoichiometric NCs show clear semiconducting electronic behaviour without showing mid-gap or deep defect states. The type III off-stoichiometric NCs (*R* = 1.08, 1.02, 0.98, 0.96, 0.89) possess mid-gap states in the bandgap. For *R* > 1, the mid-gap states are occupied whereas for *R* < 1, the states are unoccupied. For type IV NCs (*R* = 2.25, 2.08, 0.69, 0.46), metallic-like electronic behavior is observed. In addition, *E*_vac_ − *E*_F_ is also highly sensitive to the NC stoichiometry. For smaller *R*, *E*_vac_ − E_F_ is likely to decrease, suggesting a way to achieve favorable band alignments *via* controlling NC stoichiometry.

**Fig. 5:**
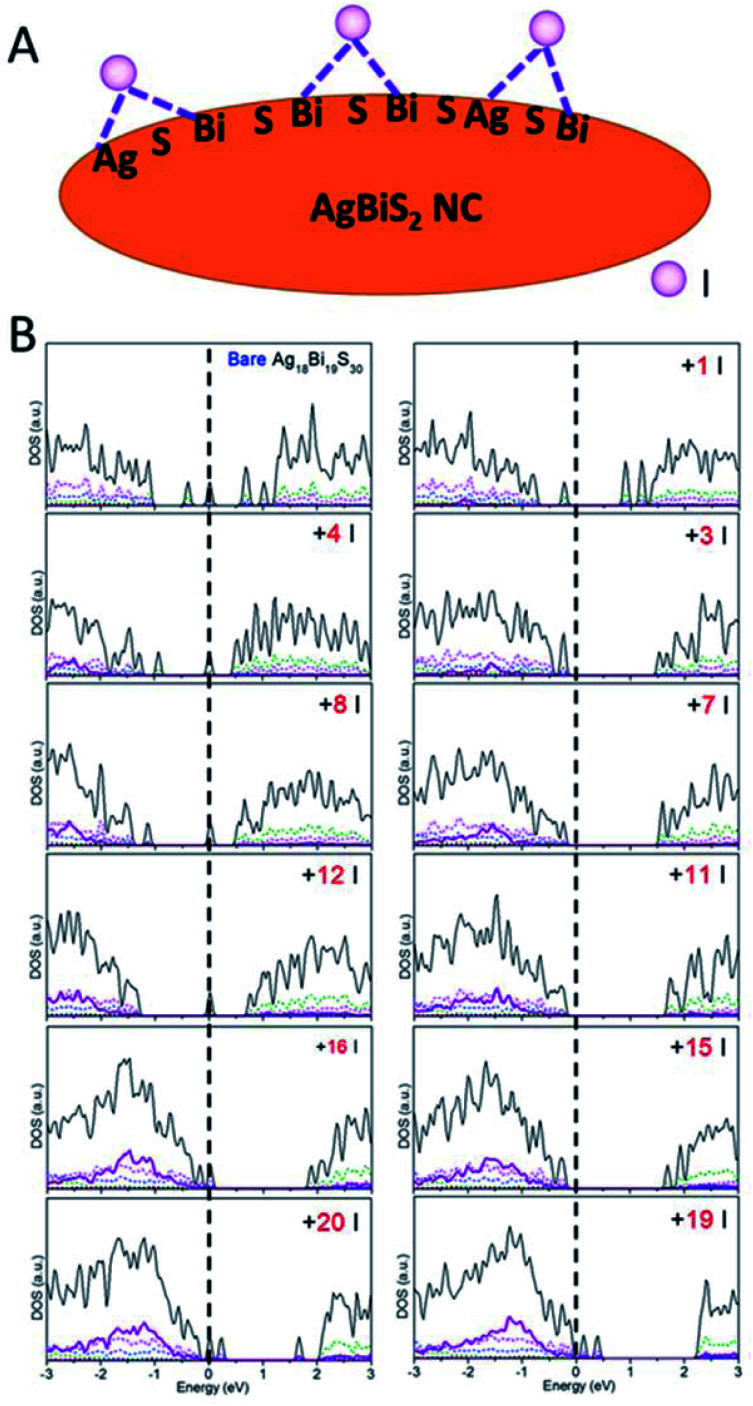
(A) Schematic of iodide ligand binding configurations on the AgBiS_2_ NC surface. (B) PDOS *versus* the number of ligands on NCs.

Besides the relationship between the stoichiometry and shape of NCs and their electronic structures discussed above, effects of ligands should be considered as well. Ligands are typically present on the NC surface during the synthesis to prevent crumpling. For AgBiS_2_, halide chemistry is a more effective passivation scheme than thiol chemistry.^1,19^ The NCs with ligand passivation can be viewed as a change in the surface stoichiometry. Here, we illustrate the effects of iodide passivation since the AgBiS_2_ NCs are generally treated with tetramethylammonium iodide (TMAI). Due to the stronger binding energy of Bi–I with respect to Ag–I, iodides tend to form covalent bonds with two surface Bi atoms or Bi and Ag atoms in the NC (see Fig. 5A).

Here, the type IV Ag_18_Bi_19_S_30_ NC was selected to explore the effects of iodide passivation. The Ag_18_Bi_19_S_30_ NC exhibits four deep-defect states, mostly localized on the corner Bi atoms and surface Bi atoms of the NC (see Fig. S16†). Fig. 5B shows the computed electronic structure *versus N*_Iodide_ (*N*_Iodide_, the number of I atoms attached to the NC). It can be seen that the Ag_18_Bi_19_S_30_ NC possesses notable stoichiometry imbalance and there are 5 excess electrons, resulting in four deep-defect states in the bandgap. We define *Δ*, as a measure of effective stoichiometry imbalance, *i.e.*, *Δ* = −*N*_Ag_ − 3*N*_Bi_ + 2*N*_S_ + *N*_Iodide_, where *N*_Ag_, *N*_Bi_ and *N*_S_ are the numbers of Ag, Bi and S atoms in the NC, respectively, and *N*_Iodide_ is the number of iodides attached to the NC. In order to make *Δ* zero, 15 iodides should be attached to the NC surface. Fig. 5B shows that the four deep defect states are remedied with the 15 iodide passivation such that the Ag_18_Bi_19_S_30_ NC is converted to a semiconductor with no deep defect states. For |*Δ*| = 3, the NC is predicted to be a semiconductor with localized mid-gap states within the bandgap, as a type III NC. For |*Δ*| ≫ 1, *e.g.*, as in the case of 1 or 20 iodide passivation, many defect states form in the bandgap. Interestingly, for |*Δ*| = 8 and 4, no deep defect states are seen in the bandgap, suggesting that only some of the excess electrons contribute to deep defect states. So the four deep defect states could be compensated with fewer iodides than 15. On the basis of these observations, the full passivation of the NC surface seems not necessarily the most desirable for optoelectronic applications. In fact, a very large |*Δ*| value can result in new deep-defect states. For example, there are new deep-defect states in the bandgap with the number of iodides increasing from 15 to 20. To achieve semiconducting electronic properties for NCs, the number of iodides used for passivation can be estimated by using the handy effective stoichiometry *Δ*. In addition, the interactions of Bi-halide and Ag-halide give rise to surface dipole moments, which changes the energy level of NCs with respect to the vacuum level, thereby tuning the band alignment of NC layers.

## Conclusions

We have performed a comprehensive study of the structure–electronic property relationship of bulk and NC AgBiS_2_. Based on the computed electronic structures of bulk structures with different orderings of Ag and Bi sites, the effect of different chains (Ag–S–Ag–S–, Bi–S–Bi–S– and Ag–S–Bi–S–) in bulk structures on the electronic property is explored. We find that the Ag–S–Ag–S– and Bi–S–Bi–S– chains contribute to the VBM and CBM, respectively. The separation of the VBM and CBM is desired for easy separation of the electron and hole carriers and benefits PV application. For AgBiS_2_ NCs, the bare NCs (types I and II) with proper stoichiometry exhibit semiconducting behaviour without showing mid-gap states or deep defect states, regardless of the NC shape. For type III NCs with slight off-stoichiometry, mid-gap states can arise in certain cases. For type IV NCs with considerable off-stoichiometry, many deep-defect states can arise in the bandgaps and some NCs even show metallic-like electronic behaviour. The effective stoichiometry imbalance established in bare AgBiS_2_ nanocrystals can be applied to ligand-passivated nanocrystals through the attachment of ligands as stoichiometry variations. We demonstrate that the deep-defect states can be removed *via* proper ligand passivation with an optimal number of ligands. The ligands can effectively render the NC-ligand system effectively stoichiometric, resulting in semiconducting behavior with no trap states for the ligand-covered NCs.

## Computational methods

### Density functional theory calculation

The first-principles computations for periodic systems are performed based on density-functional theory (DFT) methods as implemented in the Vienna *ab initio* simulation package (VASP 5.4).^27^ An energy cutoff of 400 eV is employed. The atomic positions of AgBiS_2_ bulk and NCs are optimized, using the PBEsol functional,^28^ without any symmetric restrictions until the maximum force on each atom being less than 0.02 eV Å^−1^. The ion cores are described by using the projector augmented wave (PAW) method.^29^ 12 × 12 × 8, 11 × 11 × 3 and 7 × 7 × 7 *k*-point grids are used for the AF-I, AF-II and AF-III bulk structures, respectively. A 4 × 4 × 4 *k*-point grid is used for the supercell of AF-IIb, PbS and AF-IIc bulk structures. The electronic structures of NCs are computed at the *Γ* point only, based on the more accurate HSE06 functional^30^ with a cutoff energy of 300 eV.

## Conflicts of interest

There are no conflicts to declare.

## Supplementary Material

NA-002-C9NA00505F-s001
