# Supplementary material for: AgBiS2 as a low-cost and eco-friendly all-inorganic photovoltaic material: nanoscale morphology–property relationship
Source: Nanoscale Adv. 2019 Dec 12;2(2):770–6. doi: 10.1039/c9na00505f (PMC9417815; doi:10.1039/c9na00505f)
Supplement: NA-002-C9NA00505F-s001 [file NA-002-C9NA00505F-s001.pdf]

Electronic Supplementary Material (ESI) for Nanoscale Advances.
This journal is © The Royal Society of Chemistry 2019

Electronic Supplementary Information

$AgBiS_2$ as a Low-Cost and Eco-Friendly All-Inorganic Photovoltaic Material: Nanoscale Morphology-Property Relationship

Ming-Gang Ju,[a] Jun Dai,[a] Liang Ma,[b] Yuanyuan Zhou,[c] Xiao Cheng Zeng[a,d*]

[a]Department of Chemistry, University of Nebraska-Lincoln, Lincoln, Nebraska, 68588, United States

[b]Southeast University, Nanjing 211189, China

[c]School of Engineering, Brown University, Providence, Rhode Island 02912, United States

[d]Department of Chemical & Biomolecular Engineering and Department of Mechanical & Materials Engineering, University of Nebraska-Lincoln, Lincoln, Nebraska, 68588, United States

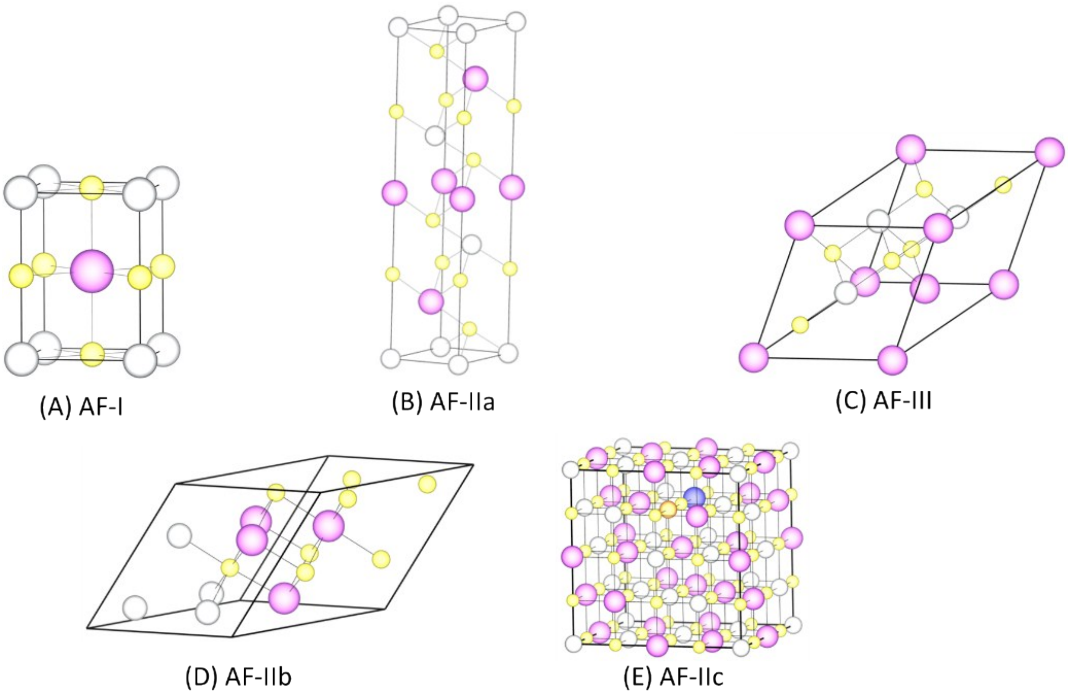

Figure S1. The primitive cells of (A) AF-I with tetragonal (P4/mmm), (B) AF-II with hexagonal representation (R$\bar{3}$m), (C) AF-III with bct (I41/amd), (D) AF-IIb with fcc (F$\bar{3}$dm) and (E) AF-IIc with monoclinic (C2/M).

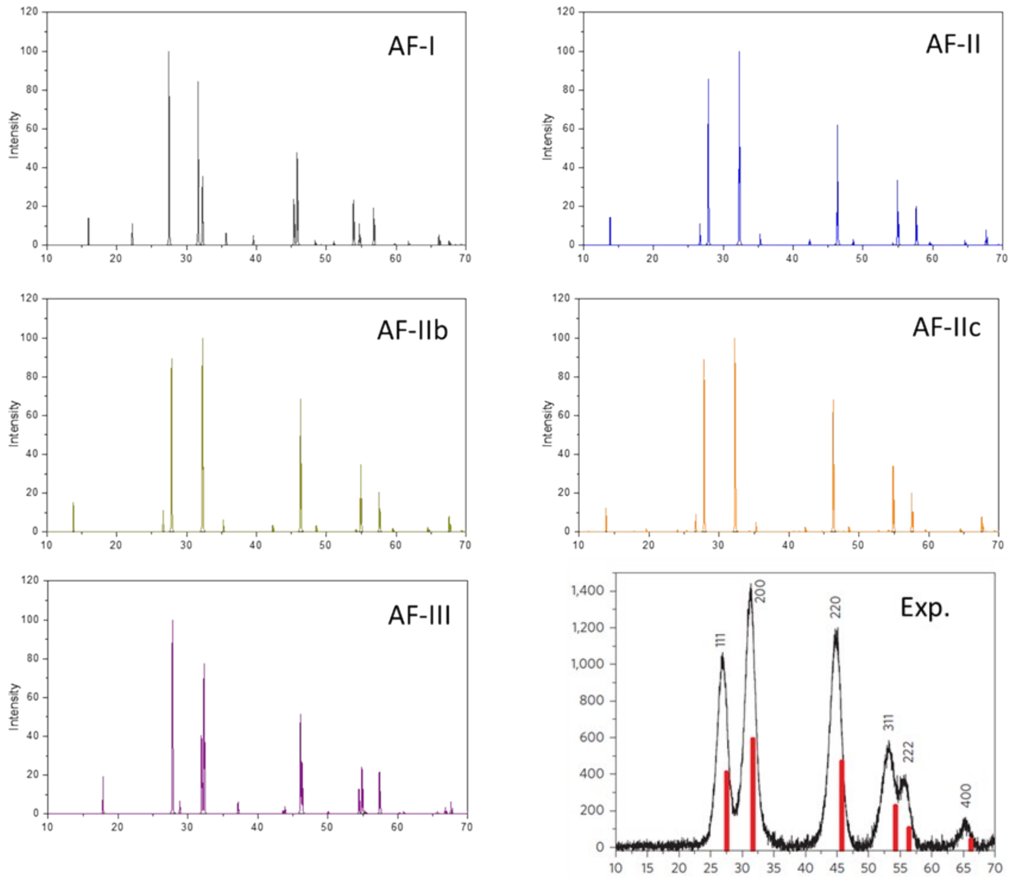

Figure S2. Simulated XRD patterns, showing intensity versus 2Θ angle, for AF-I, AF-II, AF-IIb, AF-IIc and AF-III. The experimentally measured XRD pattern is also shown for comparison.

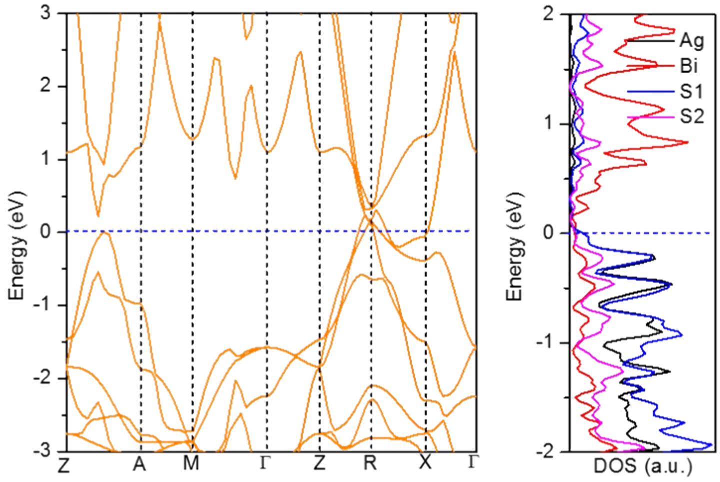

Figure S3. Computed band structure (based on the PBEsol functional) and DOS (HSE06 functional) of AF-I bulk structure.

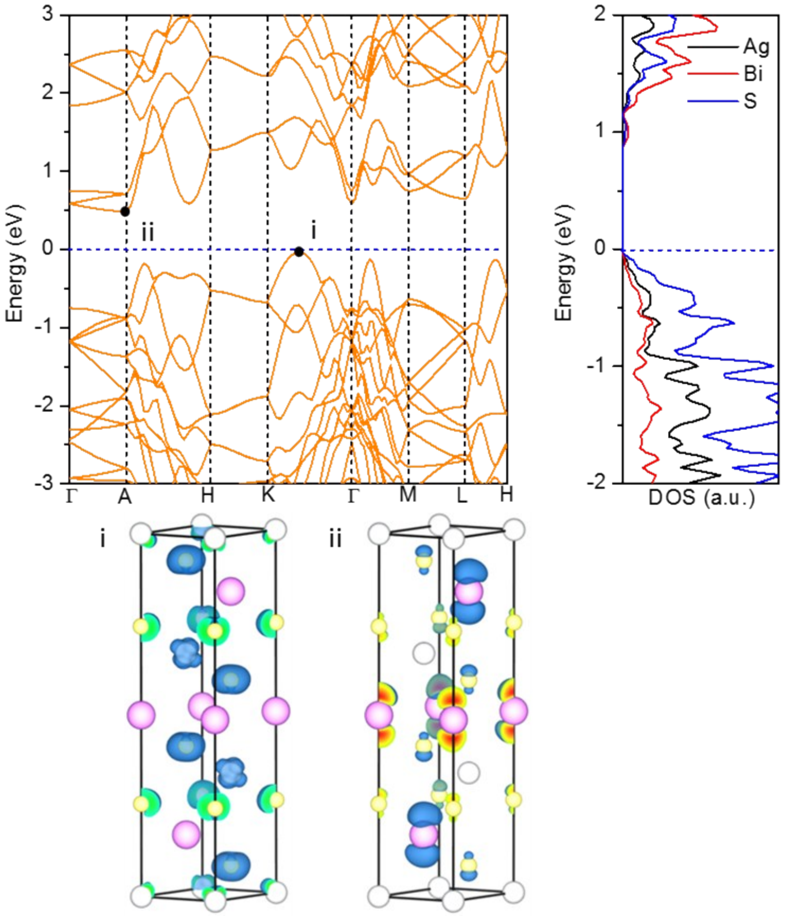

Figure S4. Computed band structure (based on the PBEsol functional) and DOS (HSE06 functional) of AF-II bulk structure. The charge density distributions of the highest VB and lowest CB of a periodic slab model of AF-II (i and ii).

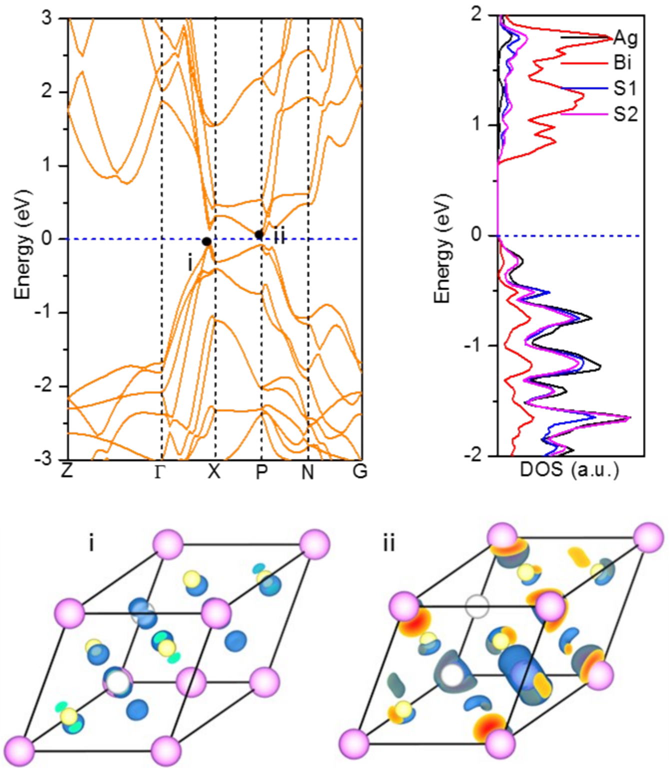

Figure S5. Computed band structure (based on the PBEsol functional) and DOS (HSE06 functional) of AF-III bulk structure. The charge density distributions of the highest VB and lowest CB of a periodic slab model of AF-III (i and ii).

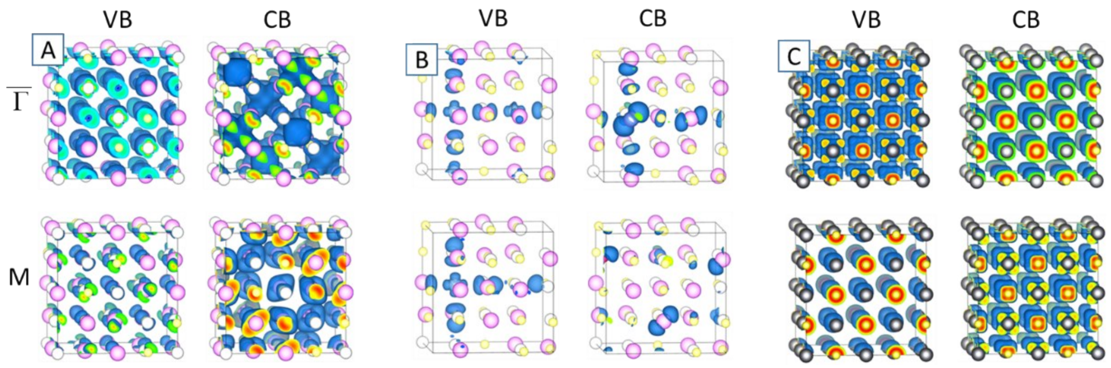

Figure S6. The spatial charge distribution of VBM and CBM at M and Γ points of AF-IIb (A), AF-IIc (B) and PbS (C).

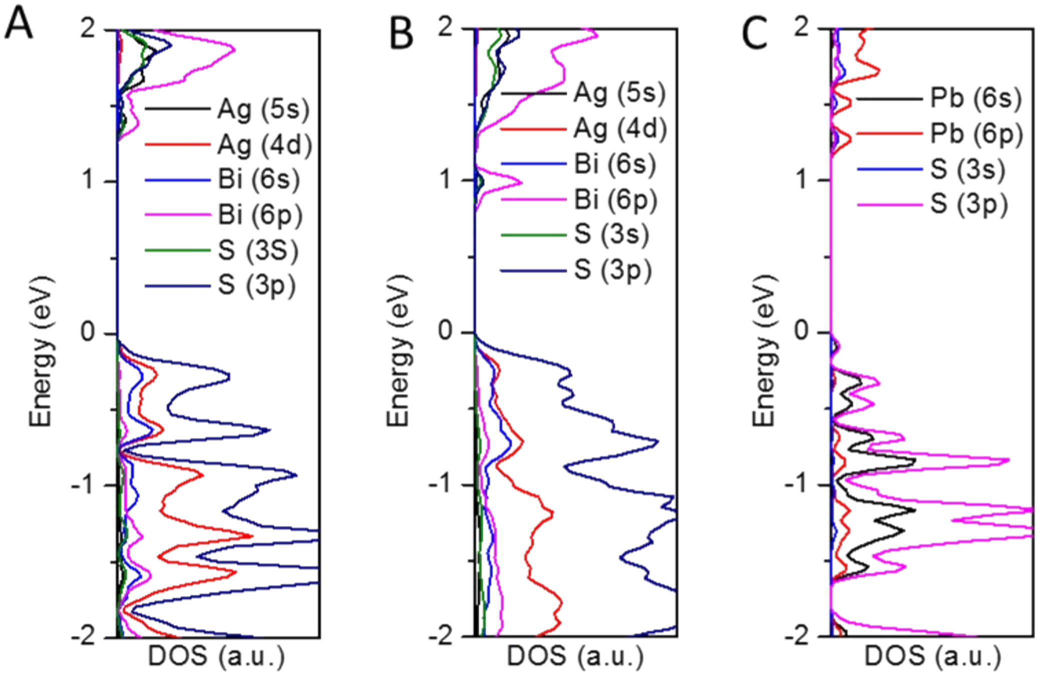

Ag

Figure S7. Computed DOSs of AF-IIb (A), AF-IIc (B) and PbS (C), based on the HSE06 functional.

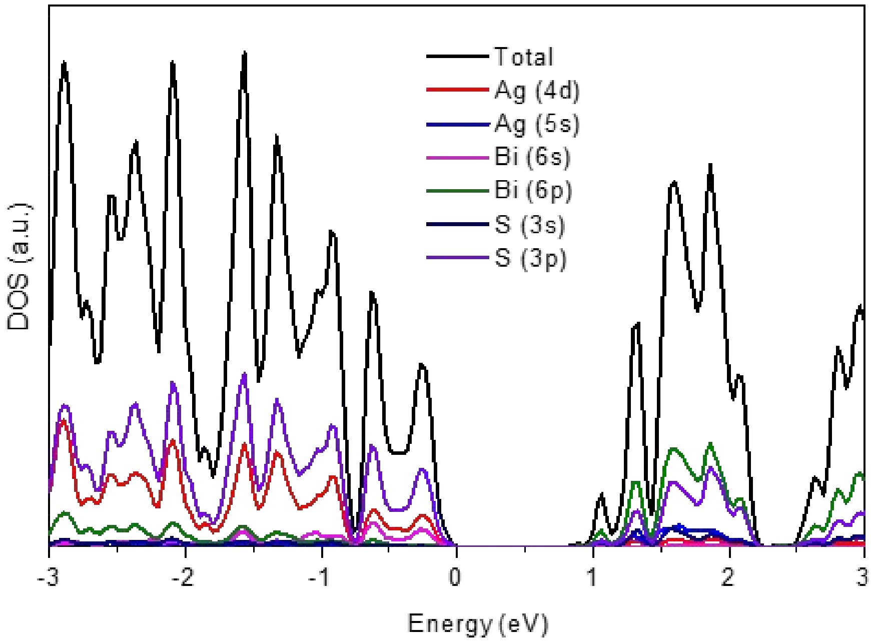

Figure S8. Computed DOSs of AF-IIb, based on the HSE06 functional with consideration of the spin-orbit coupling (SOC) effect.

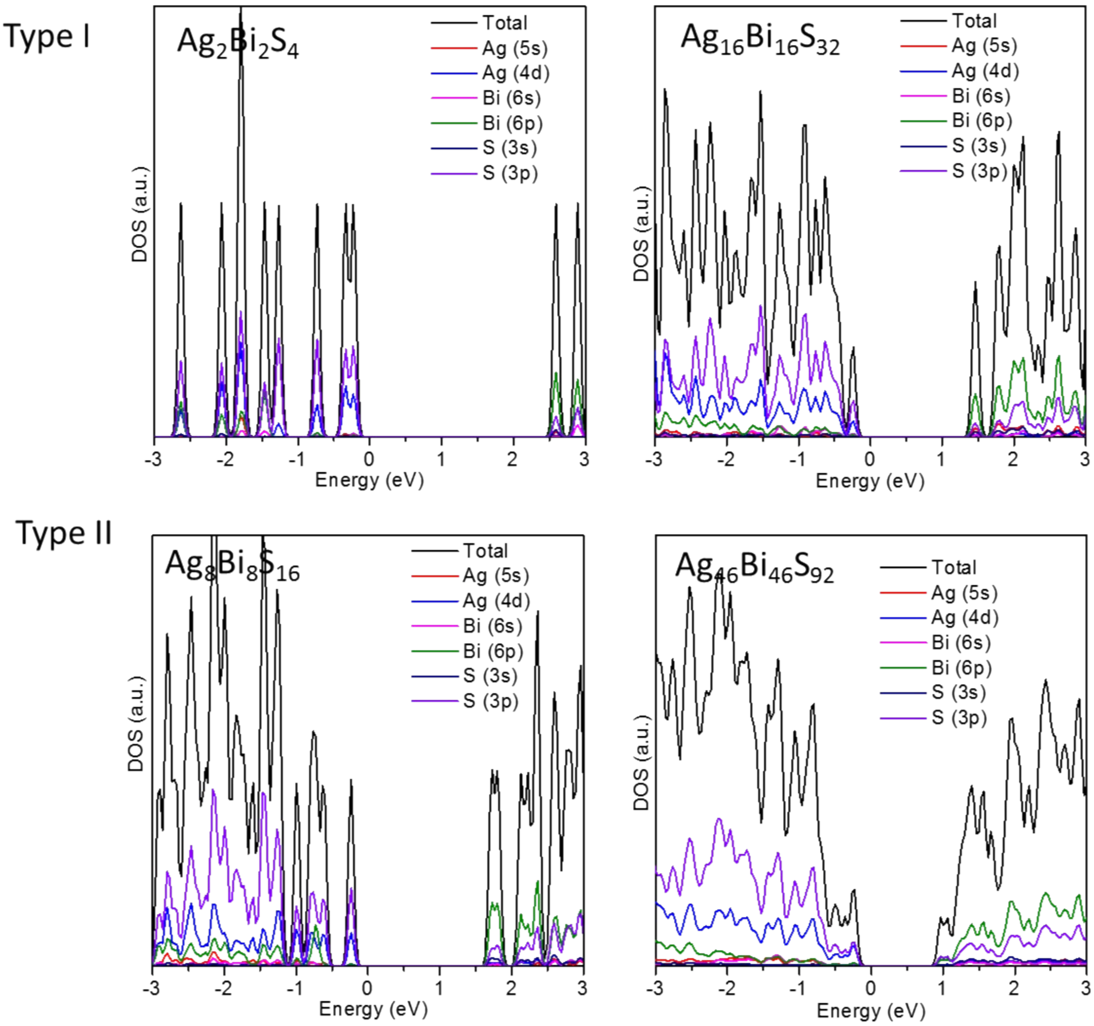

Figure S9. Computed DOSs of type I NCs $Ag_2Bi_2S_4$, $Ag_{16}Bi_{16}S_{32}$ and type II NCs $Ag_8Bi_8S_{16}$, $Ag_{46}Bi_{46}S_{92}$, based on the HSE06 functional.

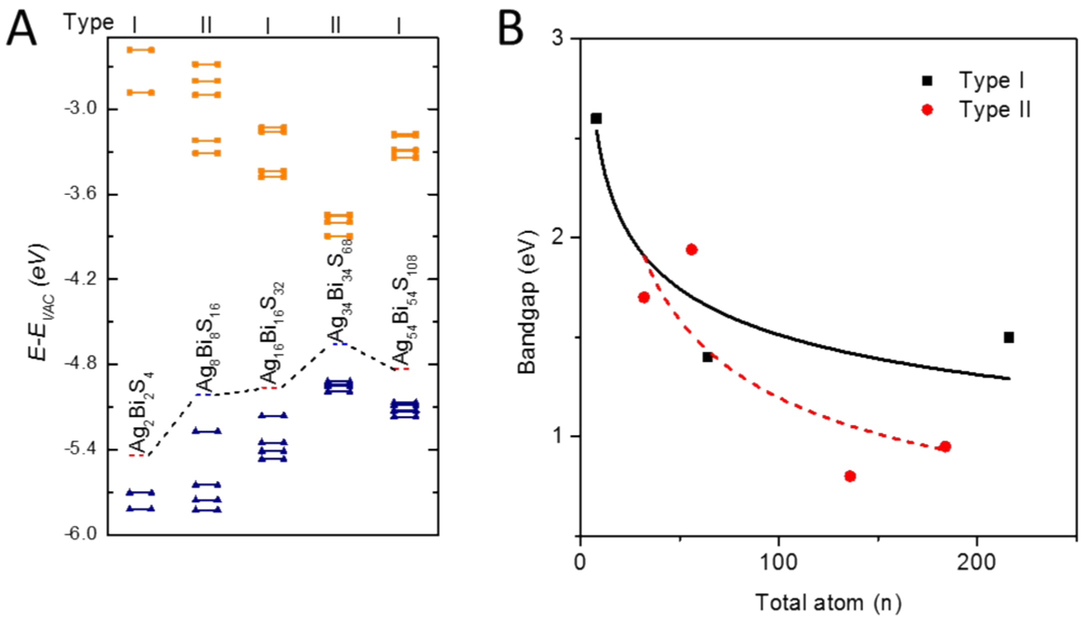

Figure S10. (A) Kohn-Sham energy levels, shifted with respect to the vacuum level, for NCs with different sizes and shapes. (B) NC size and shape effects on the band gaps of stoichiometric NCs. Quantum confinement effects cause increased band gaps for both type I and type II NCs, as their size decreases. A shape dependence is observed, suggesting slightly reduced effect of quantum confinement for the faceted cubes.

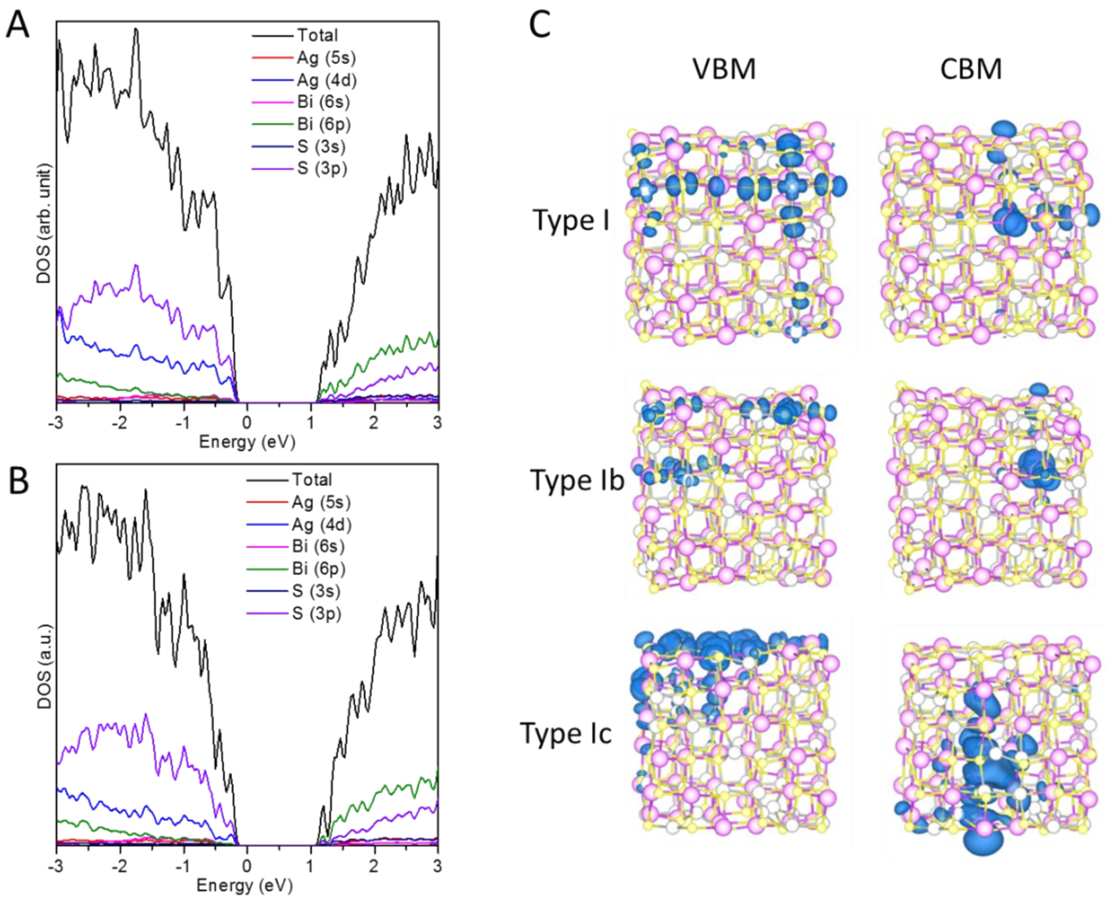

Figure S11. (A) Computed DOS of Type Ib NC with Ag and Bi sites exchanged in the NC. (B) DOS of Type Ic NC with Ag and Bi sites exchanged at the surface of NC. (C) Charge distributions of VBM and CBM corresponding to type I, type Ib and type Ic NCs, respectively.

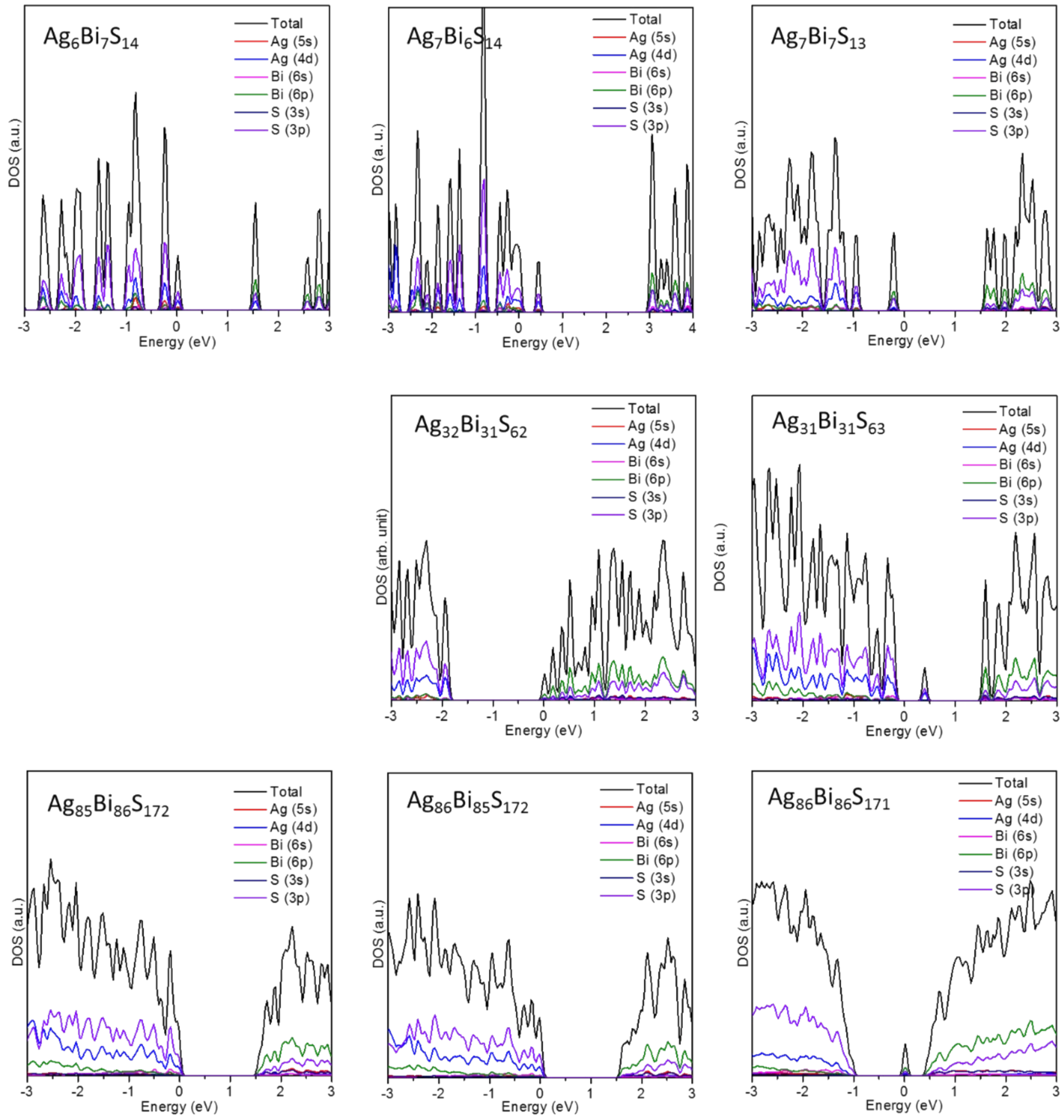

Figure S12. Computed DOSs of type III NCs $Ag_6Bi_7S_{14}$, $Ag_7Bi_6S_{14}$, $Ag_7Bi_7S_{13}$, $Ag_{32}Bi_{31}S_{62}$, $Ag_{31}Bi_{31}S_{63}$, $Ag_{85}Bi_{86}S_{172}$ and $Ag_{86}Bi_{85}S_{172}$, based on the HSE06 functional.

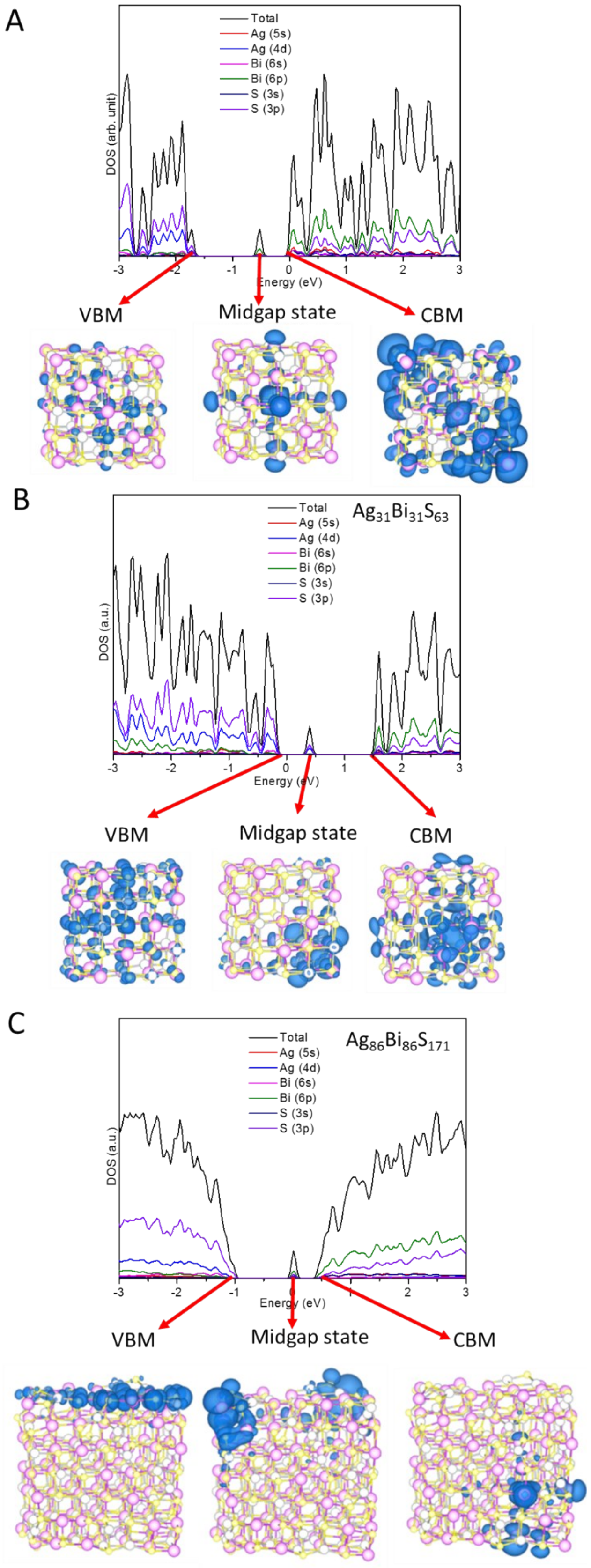

Figure S13. Charge density distribution for the states near the Fermi level in a type III $Ag_{31}Bi_{32}S_{62}$, $Ag_{31}Bi_{31}S_{63}$ and $Ag_{86}Bi_{86}S_{171}$ NCs.

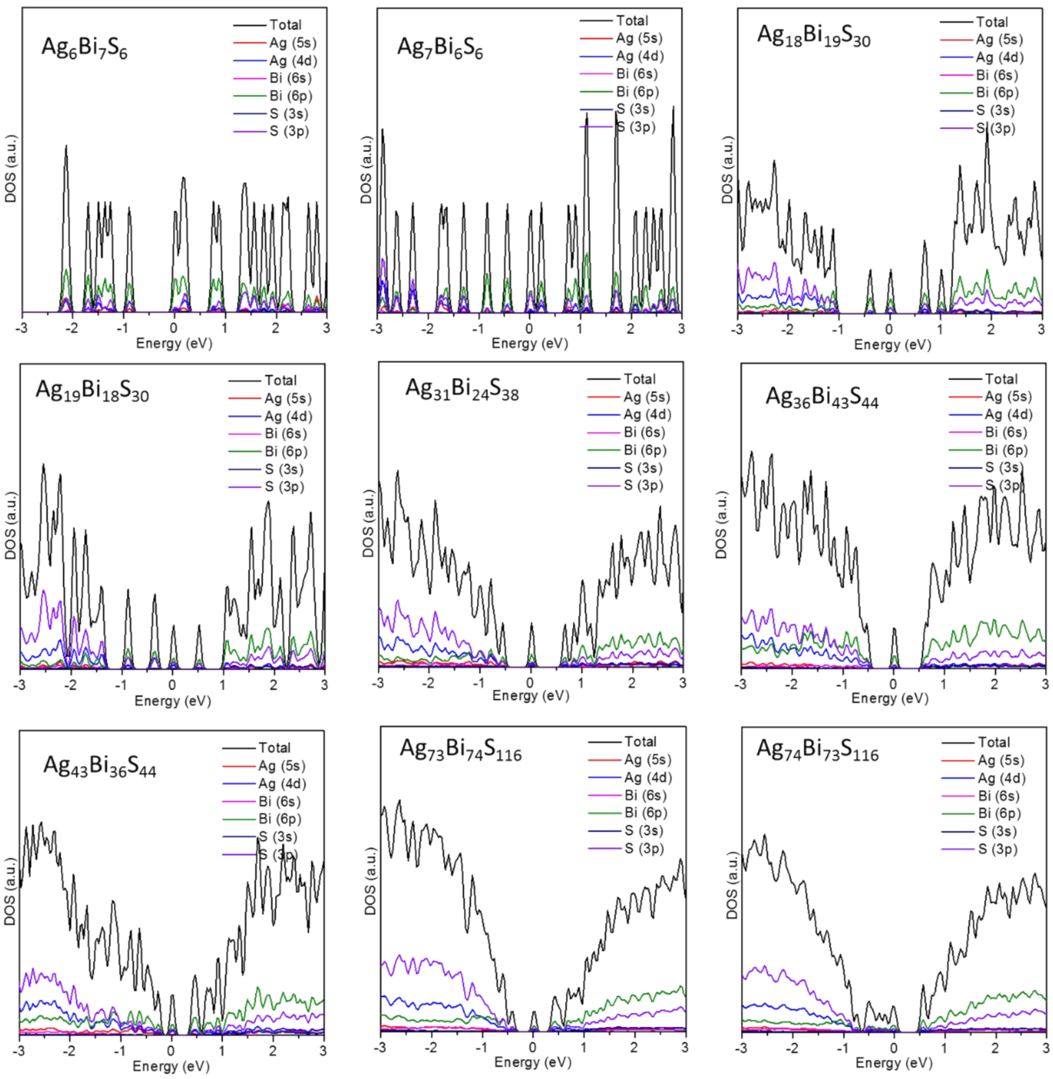

Figure S14. Computed DOSs of type IV NCs $Ag_6Bi_7S_6$, $Ag_7Bi_6S_6$, $Ag_{18}Bi_{19}S_{30}$, $Ag_{19}Bi_{18}S_{30}$, $Ag_{31}Bi_{24}S_{38}$, $Ag_{36}Bi_{43}S_{44}$, $Ag_{43}Bi_{36}S_{44}$, $Ag_{73}Bi_{74}S_{116}$ and $Ag_{74}Bi_{73}S_{116}$ with R > 1, based on the HSE06 functional.

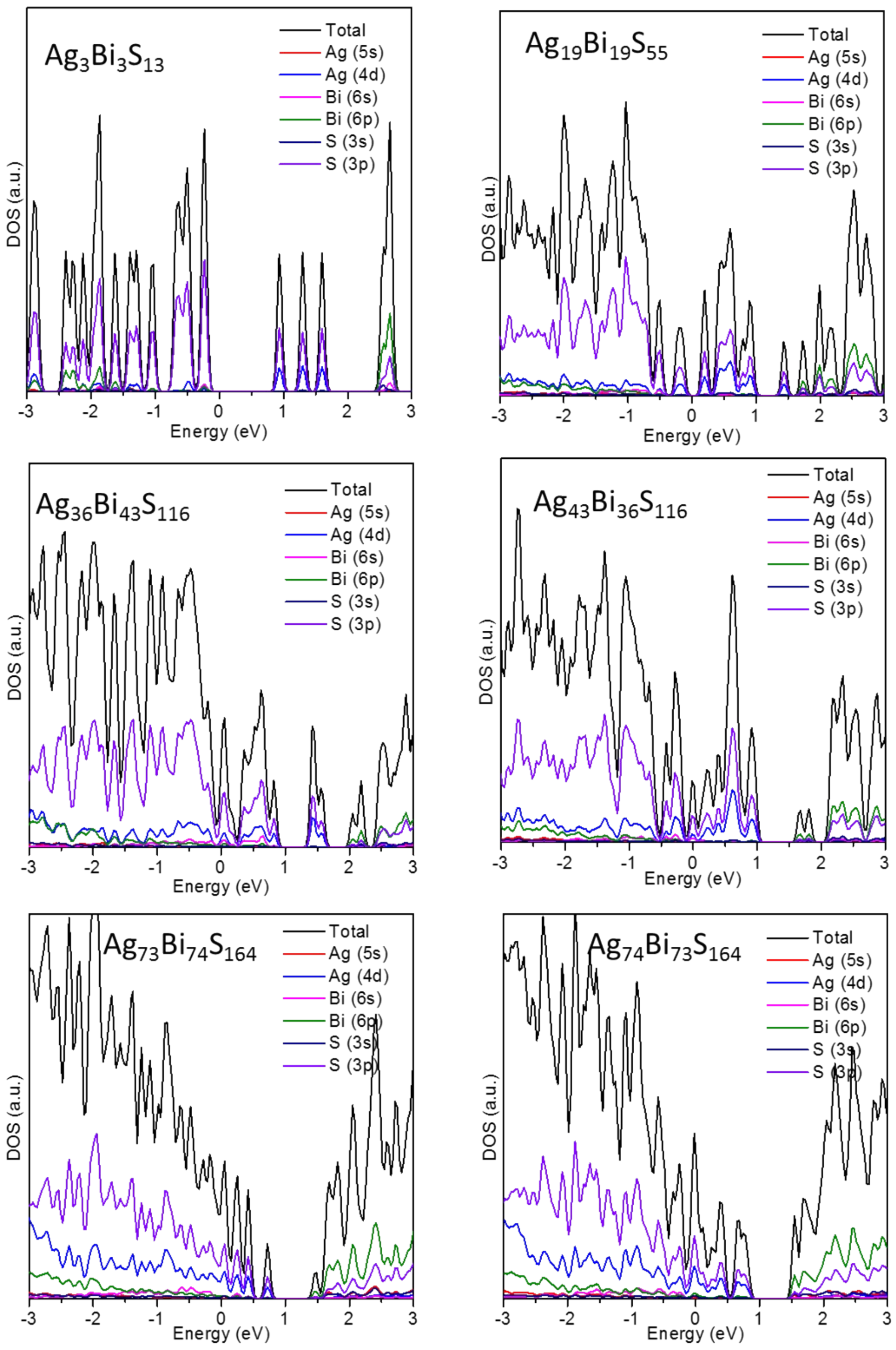

Figure S15. Computed DOSs of type IV NCs $Ag_3Bi_3S_{13}$, $Ag_{19}Bi_{19}S_{55}$, $Ag_{36}Bi_{43}S_{116}$, $Ag_{43}Bi_{36}S_{116}$, $Ag_{73}Bi_{74}S_{164}$ and $Ag_{74}Bi_{73}S_{164}$ with R < 1, based on HSE06 functional.

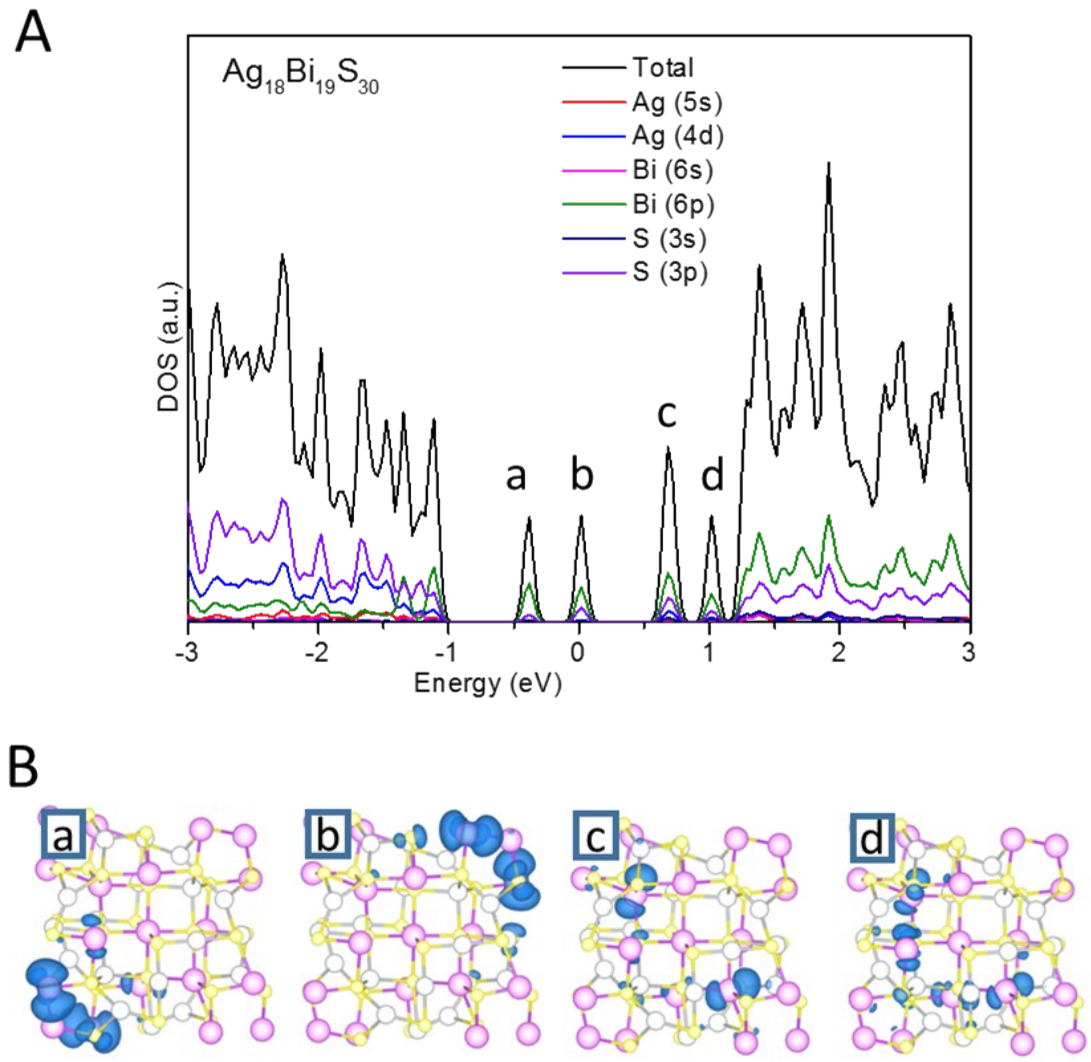

Figure S16. Charge density distribution for the deep-defect states in the bandgap of type IV $Ag_{18}Bi_{19}S_{30}$ NC.

Table S1. Computed band edge compositions of AF-IIb, AF-IIc and PbS at Γ and M points.

	AF-IIb	AF-IIc	PbS
Γ (VBM)	Ag 4d, S 3p	Ag 4d, S 3p	Pb 6s, S 3p
Γ (CBM)	Bi 6p, S 3s 3p	Bi 6p, S 3s 3p	Pb 6p, S 3s 3p
M (VBM)	S 3p	Ag 4d, S 3p	S 3p
M (CBM)	Bi 6p, S 3p	Bi 6p, S 3p	Pb 6p, S 3p
